# Lessons Learned in Sampling Community-Dwelling Black/African American Older Adults Without Advocates

**DOI:** 10.1093/geroni/igaf122.519

**Published:** 2025-12-31

**Authors:** Miriam Gofine, Antoinette Schoenthaler, Carolyn Berry

**Affiliations:** New York University, Brookline, Massachusetts, United States; New York University, New York, New York, United States; New York University, New York, New York, United States

## Abstract

“Older Adults Without Advocates” (OAWA), a clinical population referred to as “elder orphans” by the American Geriatrics Society, are conceptualized as older adults lacking kin support. While multidisciplinary literatures highlight the urgent need for more research on this growing, higher-need population, identifying OAWA in community and clinical settings remains a challenge, posing barriers to research recruitment. We report lessons learned from sampling community-dwelling Black/African American (B/AA) OAWA for a qualitative study and suggest avenues for future successes in sampling this population. Using best practices for sampling hidden populations and B/AA older adults, we utilized snowball sampling of community-dwelling B/AA OAWA with limited success. Despite strong community support for the study and recruitment, including four initial sampling “seeds,” engagement with >50 referrers across New York City to identify >60 potential OAWA, and successfully extending referral chains up to five degrees, we were only able to interview three eligible OAWA. We encountered multiple recruitment barriers, including: discrepancies between referrers’ and OAWA candidates’ perceptions of familial support; discordance between stricter clinical and looser social definitions of OAWA; gatekeepers’ reluctance or inaccessibility for referrals; inability to reach OAWA candidates using provided contact information; eligible candidates’ reluctance to participate; language barriers (Spanish and Haitian Creole); and lack of a validated OAWA screening tool. Future research should consider engaging trusted gatekeepers as interviewers, using a larger “seed” group and including OAWA residing in long-term care facilities. A validated screening tool tailored to diverse communities is essential for improving recruitment and success of research on community-dwelling OAWA.

